# FluoRender: joint freehand segmentation and visualization for many-channel fluorescence data analysis

**DOI:** 10.1186/s12859-017-1694-9

**Published:** 2017-05-26

**Authors:** Yong Wan, Hideo Otsuna, Holly A. Holman, Brig Bagley, Masayoshi Ito, A. Kelsey Lewis, Mary Colasanto, Gabrielle Kardon, Kei Ito, Charles Hansen

**Affiliations:** 1 Scientific Computing and Imaging Institute, University of Utah, Salt Lake City, USA; 2 Janelia Farm Research Campus, Howard Hughes Medical Institute, Ashburn, USA; 3 Department of Bioengineering, University of Utah, Salt Lake City, USA; 4 Institute of Molecular and Cellular Biosciences, University of Tokyo, Tokyo, Japan; 5 Department of Biology, University of Florida, Gainesville, USA; 6 Department of Human Genetics, University of Utah, Salt Lake City, USA

**Keywords:** Multichannel, Volume data, Visualization, Freehand segmentation, Analysis, GPUs, FluoRender

## Abstract

**Background:**

Image segmentation and registration techniques have enabled biologists to place large amounts of volume data from fluorescence microscopy, morphed three-dimensionally, onto a common spatial frame. Existing tools built on volume visualization pipelines for single channel or red-green-blue (RGB) channels have become inadequate for the new challenges of fluorescence microscopy. For a three-dimensional atlas of the insect nervous system, hundreds of volume channels are rendered simultaneously, whereas fluorescence intensity values from each channel need to be preserved for versatile adjustment and analysis. Although several existing tools have incorporated support of multichannel data using various strategies, the lack of a flexible design has made true many-channel visualization and analysis unavailable. The most common practice for many-channel volume data presentation is still converting and rendering pseudosurfaces, which are inaccurate for both qualitative and quantitative evaluations.

**Results:**

Here, we present an alternative design strategy that accommodates the visualization and analysis of about 100 volume channels, each of which can be interactively adjusted, selected, and segmented using freehand tools. Our multichannel visualization includes a multilevel streaming pipeline plus a triple-buffer compositing technique. Our method also preserves original fluorescence intensity values on graphics hardware, a crucial feature that allows graphics-processing-unit (GPU)-based processing for interactive data analysis, such as freehand segmentation. We have implemented the design strategies as a thorough restructuring of our original tool, FluoRender.

**Conclusion:**

The redesign of FluoRender not only maintains the existing multichannel capabilities for a greatly extended number of volume channels, but also enables new analysis functions for many-channel data from emerging biomedical-imaging techniques.

**Electronic supplementary material:**

The online version of this article (doi:10.1186/s12859-017-1694-9) contains supplementary material, which is available to authorized users.

## Background

**Fig. 1:**
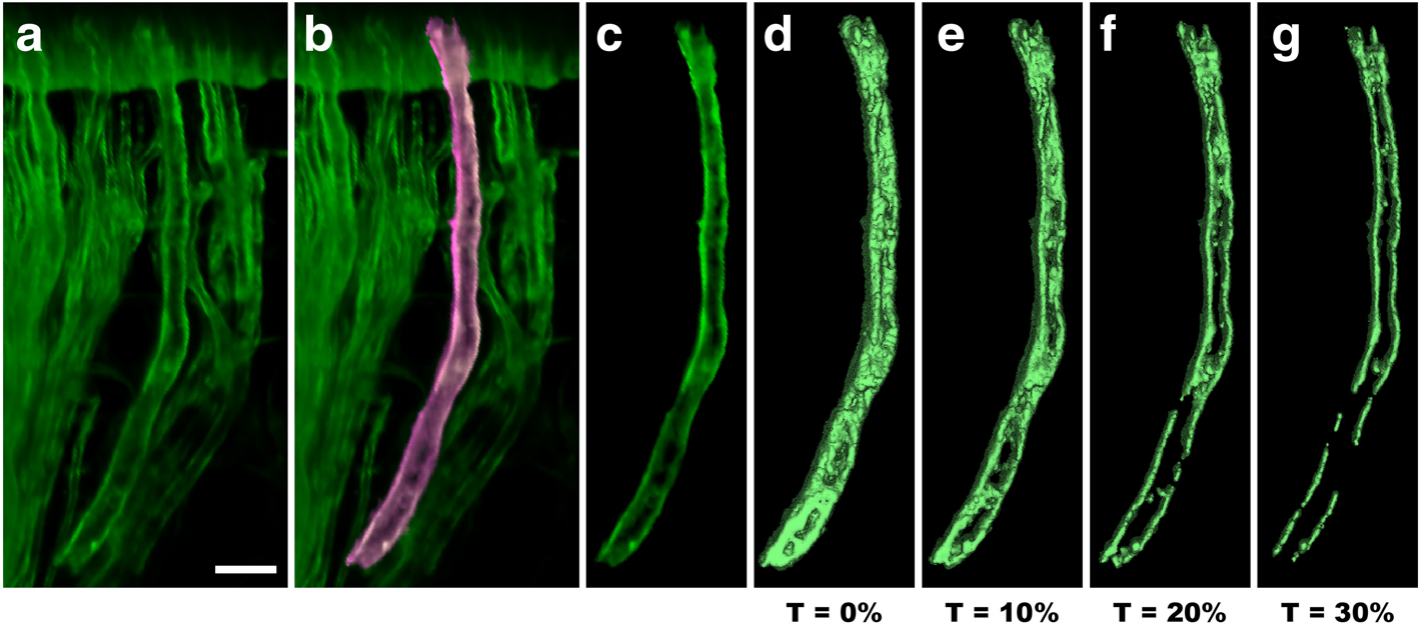
Comparison between volume-rendered and pseudosurface representations. We use FluoRender to select and extract one nerve from a confocal scan of an intact ear semicircular canal of an oyster toadfish (Opsanus tau). **a** Volume rendering of the original confocal scan. Scale bar represents 20 μm. **b** One nerve is selected and highlighted using FluoRender’s brush tool. **c** The selected nerve is segmented. **d** The selected nerve is converted to a pseudosurface representation, using the marching cubes algorithm with a threshold at 0% grayscale. **e** A pseudosurface representation with a threshold at 10% grayscale. **f** A pseudosurface representation with a threshold at 20% grayscale. **g** A pseudosurface representation with a threshold at 30% grayscale. The result shows that conversion of volume data to pseudosurfaces is not suitable for visualizing intensity variations, which are essential for fluorescence microscopy. The parameter settings for pseudosurface conversion may greatly influence the results. Subsequent analysis and comparison based on pseudosurfaces may generate misleading results

Recent research on the insect nervous system has developed data processing techniques for image registration and segmentation, which enable us to place large amounts of volume data, morphed three-dimensionally, onto a common spatial frame, called a template, for visual examination and computational analysis [1, 2]. In such applications, several tens of independent three-dimensional (3D) structures need to be visualized and analyzed simultaneously in an anatomical atlas. To preserve the fine details of the nervous system, structures represented by volume data with varying intensity values are preferred to polygon-based geometry data. The geometry data, also called pseudosurfaces, can be easily rendered, combined, and manipulated with decent computer hardware. However, details of the original data are either compromised or replaced with spurious geometries. It is difficult for a pseudosurface to represent the intensity variations embedded within the original grayscale volumes. Choosing a criterion for generating pseudosurfaces from ill-defined structural boundaries can be challenging. Figure 1 compares volume-rendered and pseudosurface representations of one nerve extracted from a confocal scan of an intact ear semicircular canal of an oyster toadfish (*Opsanus tau*). No matter how carefully we choose the threshold values for pseudosurfacing, the fact that the center of the nerve expresses less fluorescent reporter protein is obscured. Atlases composed of such pseudosurfaces can become unreliable and misleading, especially when fine details need to be quantitatively analyzed and compared.

To facilitate volume-based atlas building and neural structure analysis, we have developed a paintbrush tool in FluoRender [3], a freehand tool that allows users to paint directly on visualization results using a mouse or digital stylus, with convenient viewing directions for the best display of a structure [4]. The strokes painted two-dimensionally are used to select and segment grayscale values in 3D. The tool has been used in biological research since its introduction [5–8]. It solved the issues of pseudosurfacing by adopting a workflow based entirely on channels, sometimes also called layers, which include spectrally distinct channels, subvolumes derived from segmentation, or coregistered data sets from different scans. The capability to intuitively select, extract, label, and measure multiple biological structures from 3D visualizations is also instrumental to higher-level analysis workflows. For example, colocalization analysis and morphological comparison of several neural structures benefit from isolating these structures and cleaning up background signals; tracking 3D movements of cells often requires focusing on one or several subsets for detailed studies or troubleshooting issues from automatic algorithms.

Our tool generates new volume channels from selected and extracted structures. The freehand segmentation can be performed on a sequence of coregistered scans or on a single scan that contains overlapping structures because of limited Z-slice resolution. Therefore, the introduction of freehand segmentation tools requires simultaneous support of an increasing number of volume channels from interactive data analysis workflows, which can be challenging for both rendering and data processing. Unfortunately, current bioimaging software tools are ill equipped to interactively visualize or analyze a large number of volume channels. Although there have been techniques and tools that support large-scale data of high spatial resolutions [9–11], most of them have fallen short when the number of channels increases. Researchers often overlook multichannel as one important source of big data. In the past, a 3D scan was comprised of a single grayscale channel, because research on volume data handling originated from X-ray computed tomography (CT) [12], which generated only one channel. To date, 3D imaging tools that can directly handle more than three channels are scarce. The commonly used ImageJ [13] and Fiji [14] can store multiple channels with the “hyperstack” feature, but cannot visualize them three-dimensionally. The Visualization Toolkit (VTK) [15] does not provide a module for more-than-RGB channel visualization. Thus, it becomes difficult for the software tools dependent on VTK (OsiriX [16], 3D Slicer [17], Icy [18], and BioImageXD [19]) to fully support multichannel data from fluorescence microscopy. There are tools for visualizing multiple channels with only RGB channels, which are commonly supported by graphics hardware. They use a preprocessing approach to blend and map data channels into the RGB space and then render the derived color channels [9, 20]. This approach is adopted for various mainstream visualization and analysis tools for biological research. For example, the commercial software package, Amira [21], and the nonprofit tool, Vaa3D [9, 22], both adopt a preprocessing routine to first combine multiple grayscale volumes into one RGB volume and then render the combined volume. An inconvenience of combining channels during preprocessing is that any adjustment to the visualization parameters of one channel requires a recalculation of the entire blended volume, making it difficult to tune the appearance of the visualization interactively. Another commercial software package, Imaris [23], mitigates this issue by postponing the channel combination process until rendering. However, its implementation attaches channels at different texture-mapping units (components within the graphics hardware to address and filter images [24]) all at once, thus limiting the total number of channels as a result of the available texture units or graphics memory size. The complexity of handling multiple texture units also prevents volume-based interactive analysis on GPUs. (Details are in the survey in the Additional file 1: Supplementary methods and results.)

In the biomedical sciences, a versatile tool allowing visualization-based analysis for many-channel data is crucial for researchers to decipher fluorescence microscopy scans, perform qualitative and quantitative data analysis, and present outcomes. The challenges are twofold from a software development perspective. 1) Volumes from a many-channel data set need to be combined or intermixed for viewing, but intensity values of each channel need to be preserved for interactive adjustment, editing, and analysis. These requirements minimize the benefits of preprocessing in Amira and Vaa3D as channel data processing becomes dynamic and dependent on user inputs. 2) Freehand analysis tools, such as the paintbrush in FluoRender, require real-time processing, which has been achieved by using GPUs. However, hardware resources within a GPU are limited, making it impractical to use many texture units, as in Imaris. It is essential to rethink and redesign the basic data model for handling channels for accurate, efficient, and versatile visualization-analysis workflows. Here, we present a thorough reconstruction of our original software tool, FluoRender [3], achieving a joint freehand segmentation and visualization tool for many-channel fluorescence data. Compared to the original tool, which supported fewer than 10 channels, the upgrade significantly extends the capacity for multichannel data and addresses the two challenges in a many-channel setting. The new FluoRender uses the latest graphics application programming interfaces (APIs) to integrate intuitive analysis functions with interactive visualization for multichannel data and ensures future extensions for sophisticated visualization-based data analysis.

## Implementation

As discussed in the Results, our design of FluoRender enables a true multichannel pipeline to visualize, segment, and analyze 3D data from fluorescence microscopy, supporting far more than three RGB channels. To support the extended multichannel capacity as well as maintain the existing visualization features, such as channel intermixing, our implementation is a reorganization of the original rendering and processing pipelines built on top of a novel multilevel streaming method. For the sake of simplicity, the discussion is organized into thematic topics that parallel those in the Results, so that readers may cross-reference the related topics from both sections.

## Multichannel streaming

**Fig. 2:**
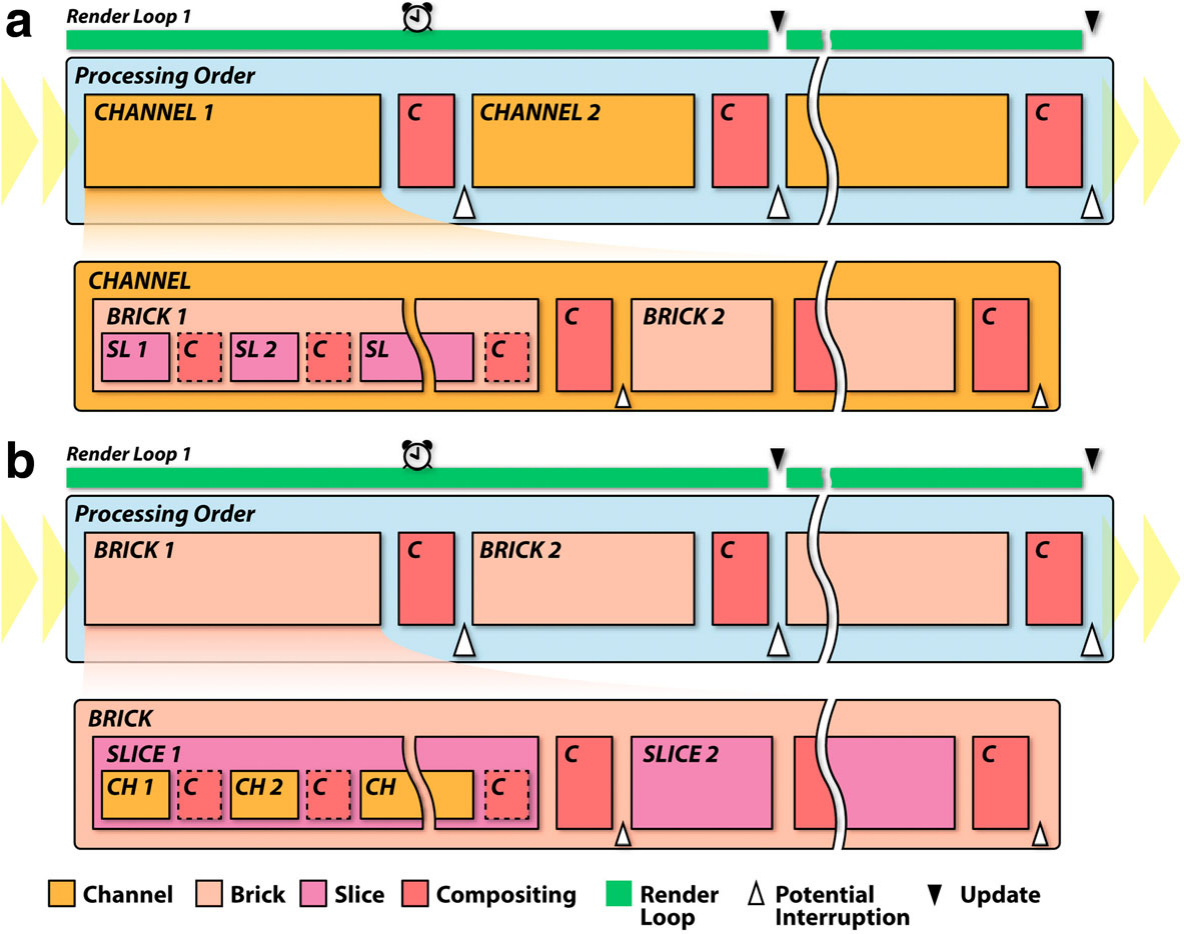
Different channel-intermixing modes use two data streaming orders for multichannel data. **a** For the layered and composite-intermixing modes, channels are streamed at the highest level. In this level, each channel is rendered and composited in sequence. Within the level of each channel, bricks are rendered in sequence. Then, within each brick, slices are rendered in sequence. **b** For the depth channel-intermixing mode, the streaming order is shifted, where bricks are at the highest level. Within each brick, slices are rendered in sequence. Then, within each slice, channels are rendered in sequence. By shifting the order of data streaming and applying different compositing methods, a variety of rendering effects become available

A many-channel data set can be considered as extremely high information density with a large number of intensity values colocated at each voxel. The streaming method, which processes only a portion at a time of a large data set that cannot fit within the graphics memory or GPU processing power, is adopted for interactive presentation. However, unlike large data of high spatial resolutions, when a many-channel data set is relatively small for each channel, the multiresolution streaming method becomes ineffective (e.g., Vaa3D [22]). For example, a 100-channel data set requires downsampling each channel 100 times to achieve an interactivity similar to that is achieved by rendering just one channel. The downsampled results may become too blurry to be useful for any analysis. Therefore, for streaming many-channel data, we adopted a different hierarchy with three levels of channels, bricks, and slices (Fig. 2).

First, a many-channel data set is naturally divided into channels. Depending on the data’s spatial resolution and user settings, each channel is then subdivided into axis-aligned subvolumes, called bricks. Finally, each brick is decomposed into a series of planar sections, called slices. The decomposition of a brick into slices is computed interactively with the viewing angle, so that each slice is always facing the observer to minimize slicing artifacts. Channels, bricks, and slices define three hierarchically processed levels of the streamed rendering. We allow users to choose an interaction speed in terms of the milliseconds allocated to render a frame. FluoRender then calculates the amount of data that can be processed and rendered within the time limit. For a many-channel data set, such as the *Drosophila* brain atlas in the Results, updates can be progressive. However, FluoRender allows great flexibility for interactive adjustments. We designed the system so that all computations are executed by parallel processing on GPUs. By using the modern OpenGL visualization pipeline [24, 25], the system can benefit from the latest technical advances of GPUs. Visualized volume data can be translated and rotated in real time; any change in a channel visualization setting is reflected interactively.

## Visualization of channel data

We use a slice-based renderer to visualize one channel in a many-channel data set and allow its flexible adjustments. Not only is a slice-based renderer more suitable for our data streaming hierarchy, the finest level of which consists of slices, but it also is more versatile than another commonly used method: ray casting [26]. A ray caster generates rays from the viewer and samples them within the volume. The sample values are retrieved sequentially along each ray and integrated based on a compositing equation. The final output is a 2D image composed of integration results from the rays. Using modern graphics hardware, the computation for each ray can be carried out in parallel, allowing real-time visualization. A slice-based renderer decomposes a volume into a series of planar sections parallel to each other, sequentially renders each section, and then composites the rendered results. Different from ray casting, slice-based rendering of the sample points on each section can be carried out in parallel. When the slicing angle is calculated in real time to be perpendicular to the viewing direction, results from both methods are similar in terms of rendering speed and quality. However, when handling more than one volume channel, a ray caster needs to sample all channels before the ray integration can proceed in the sequential sampling process. Therefore, on graphics hardware, ray casting requires all channels to be loaded and bound to available texture units, which become its limitation. In contrast, a slice-based renderer sequentially processes an identical planar section for different channels, composites the results, and then proceeds to the next section. It is then possible to serialize the processing of multiple channels and remove the limitation on the total number of channels. A second limiting factor for the ray caster is sending the control information for all channels, such as parameters for color mapping, opacity mapping, etc. A ray caster not only requires all the available texture units, but also that all the control information be sent and processed at the same time. These requirements can severely limit the number of adjustments one channel may have. Otherwise, the rendering code becomes too complex to manage all settings from all channels. The choice of the slice-based rendering method in FluoRender allows an abundance of settings for each channel.

We maintained the existing versatile visualization configurations of the original FluoRender system and extended them for many-channel applications. In a many-channel data set, independent channel adjustment and multiple options can be applied to render each channel. For the base rendering modes, FluoRender offers two major rendering methods. The direct volume rendering (DVR) method requires high computational loads but generates realistic 3D images that reflect the physics of light transmission and absorption [26, 27]. The second method, maximum intensity projection (MIP), is much simpler. This method takes the maximum value of the signal intensities among the voxels that are along the light path viewed by the observer [28]. In addition to the two base modes, users have options to add color, a color map, Gamma, contrast, depth effect, transparency, and shading and shadows for each channel [29].

## Channel intermixing

FluoRender handles the visualization of each channel independently, allowing a mixture of different volume rendering modes and settings in a single visualization. The updated FluoRender inherited the three channel-intermixing modes from the original system [3]. The depth mode intermixes channels with respect to their perceived depth along the viewing direction; the composite mode accumulates the intensity values of individually rendered channels; and the layered mode superimposes individually rendered channels on top of one another.

One challenge for the new system is to faithfully support these channel-intermixing modes in the many-channel setting. It is crucial to have the correct streaming order for the desired channel-intermixing results. We designed two streaming orders by shifting the hierarchical levels in which channels, bricks, and slices are processed. In the layered and composite channel-intermixing modes, channels are processed at the highest level, bricks at the second, and then slices (Fig. 2a). In the depth channel-intermixing mode, bricks are processed at the highest level, slices at the second, and then channels (Fig. 2b). For a long processing sequence, the entire streaming process is allocated into several render loops (green stripes in Fig. 2), each consuming a predefined amount of time (alarm clocks in Fig. 2) and processing only a portion of the entire sequence. To prevent the system from becoming unresponsive, the visualization result is updated between two render loops (black triangles in Fig. 2). Users are also allowed to interrupt the process at certain points of the sequence (white triangles in Fig. 2) for good interactivity. Memory size, brick size, and system response time are adjusted for different hardware configurations in system settings.

**Fig. 3:**
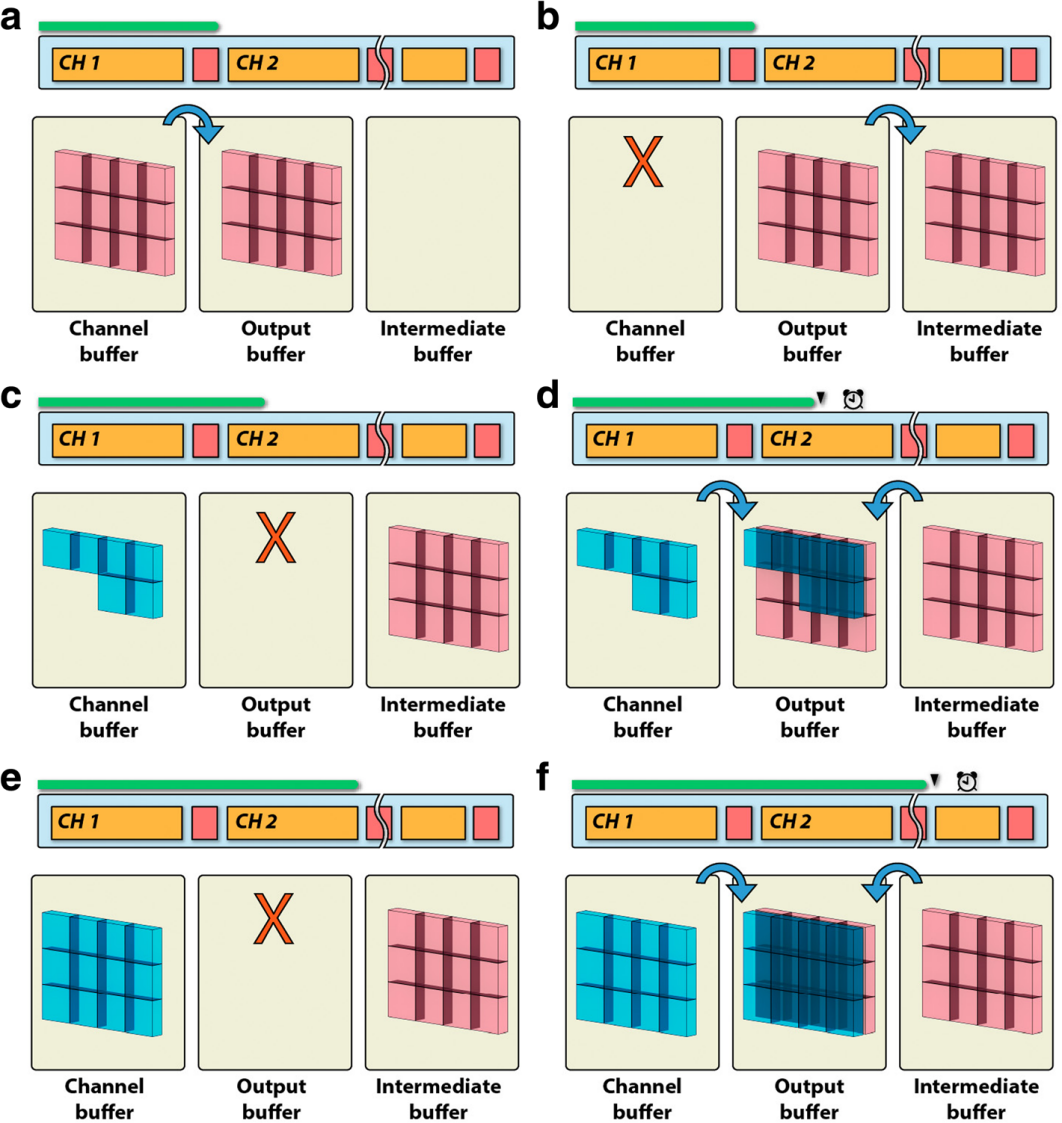
The triple-buffer rendering scheme ensures that different compositing operations are free from interference. In this example, two channels are rendered using DVR, and then intermixed in the composite mode. The panels are the steps for processing the channels. **a** Channel 1 (*red*) finishes rendering to the channel buffer. Its result is copied to the output buffer. **b** The channel buffer is cleared; the content of the output buffer is copied to the intermediate buffer. **c** A portion of the bricks of Channel 2 (*blue*) is rendered; the output buffer is cleared. **d** The results in the channel and intermediate buffers are composited together to the output buffer, which is then shown to users. Render loop 1 finishes. **e** Rendering of Channel 2 continues and finishes. The output buffer is cleared. **f** The results in the channel and intermediate buffers are composited together to the output buffer. This process is repeated when more than two channels are present

The second challenge is to support a variety of compositing operations (red squares in Fig. 2), as each channel can be configured differently according to its render settings. Table 1 summarizes all compositing methods in FluoRender. To avoid the interference of different compositing methods from different hierarchical levels within the streaming process, a triple-buffer rendering scheme is adopted. Figure 3 illustrates an example of using three buffers (channel, output, and intermediate) in the streaming process to generate the correct result of two channels intermixed with the composite channel-intermixing mode. In this example, the tri-buffer rendering is necessary because rendering one channel uses the front-to-back compositing whereas the composite mode uses the addition compositing (Table 1). As the buffers use completely different compositing equations, partial results cannot be intermixed correctly when fewer than three buffers are used in the streaming process. An intermediate buffer is employed to temporarily store the rendering results from completed channels, each of which uses the same compositing. The rendering and compositing of the partial result from an ongoing channel is effectively isolated from the compositing between channels. Therefore, FluoRender is able to support versatile visualization configurations for multiple channels.

**Table 1:** Compositing methods in FluoRender

Type	Equation^a^	Description	Use
Front-to-back	${C}_{out}=\left(1-{\alpha}_{dest}\right)\bullet {C}_{source}+{C}_{dest}$	Blends semitransparent layers from front to back. Also used for ray casting	Direct volume rendering (DVR) for one channel
Back-to-front	${C}_{out}={\alpha}_{source}\bullet {C}_{source}+\left(1-{\alpha}_{source}\right)\bullet {C}_{dest}$	Blends semitransparent layers from back to front	Layered channel- intermixing mode
Addition	${C}_{out}={C}_{source}+{C}_{dest}$	Sums input and existing intensity values	Composite channel- intermixing mode; compositing operations between slices in depth mode; visualization of selected structures
Maximum intensity	${C}_{out}= MAX\left({C}_{source},{C}_{dest}\right)$	Finds the maximum intensity value from the input and existing values	MIP rendering for one channel
Multiplication	${C}_{out}={C}_{source}\bullet {C}_{dest}$	Multiplies the intensity value of the input by the existing value. Also called modulation	Shading and shadow effects

^a^In the equations, ${C}_{out}$, ${C}_{source}$, and ${C}_{dest}$ denote the output, input, and existing color values, respectively; $\alpha$ is the opacity value

## Floating-point rendering

A danger of intermixing intensity values from many channels is signal clipping of the accumulated result, where the intensity of colocalized structures exceeds the limit that can be reproduced by display hardware, causing loss of details. To address this problem, we use 32-bit floating-point numbers [24, 30] for the composite buffer through the rendering process, which preserves the high-intensity details without clipping. Using 32-bit floating-point numbers also takes advantage of recent display devices featuring 10-bit intensity resolution (30-bit for RGB) [31, 32]. FluoRender is able to directly utilize the higher color/intensity resolving power of the latest display systems for biological research and applications.

Floating-point calculation generates image data that often contain pixels whose intensity is above the clipping threshold of the display device. Such an image is called a high dynamic range image (HDRI) [33]. Instead of clipping the values at the threshold, a tone-mapping curve can be applied to normalize the full range of output intensity into that supported by the display device, so that fine details of the high-intensity regions are recovered. For an easy control of the complex tone-mapping process, we designed three adjustable parameters: Luminance scales overall intensity uniformly; Gamma changes contrast by adjusting mid-tone levels; and equalization suppresses high- intensity values and enhances low ones, thus equalizing the brightness [29].

## Freehand segmentation

Fluorescence microscopy data tend to contain signals of multiple cells and structures. Specific subparts need to be extracted, or segmented, for selective visualization and quantitative analysis. However, the data discrepancy between the visualization (pseudosurfaces or blended channels) and original grayscale channels has made channel segmentation nonintuitive in other tools, and analysis based on pseudosurfaces or blended channels inaccurate. In our multichannel design of FluoRender, data to be visualized and analyzed are essentially the same, enabling seamless operations with GPUs for both rendering and general computing [34]. Many of FluoRender’s analysis functions depend on this unique feature to directly select subvolumes of grayscale values using a brush tool. A subvolume for a focused study of isolated biological structures is also called a region of interest (ROI). Traditional ROI selection methods, such as 3D clipping planes, generate straight and arbitrary boundaries, which are not ideal for precise analysis. For example, a statistical analysis can be biased by a careless selection of an ROI, including excessive background signals. For intuitive selection of an ROI that is pertinent only to the biological structures under study, or an SOI (structure of interest), a 3D mask based on signal intensity and distribution can be generated by freehand selection.

**Fig. 4:**
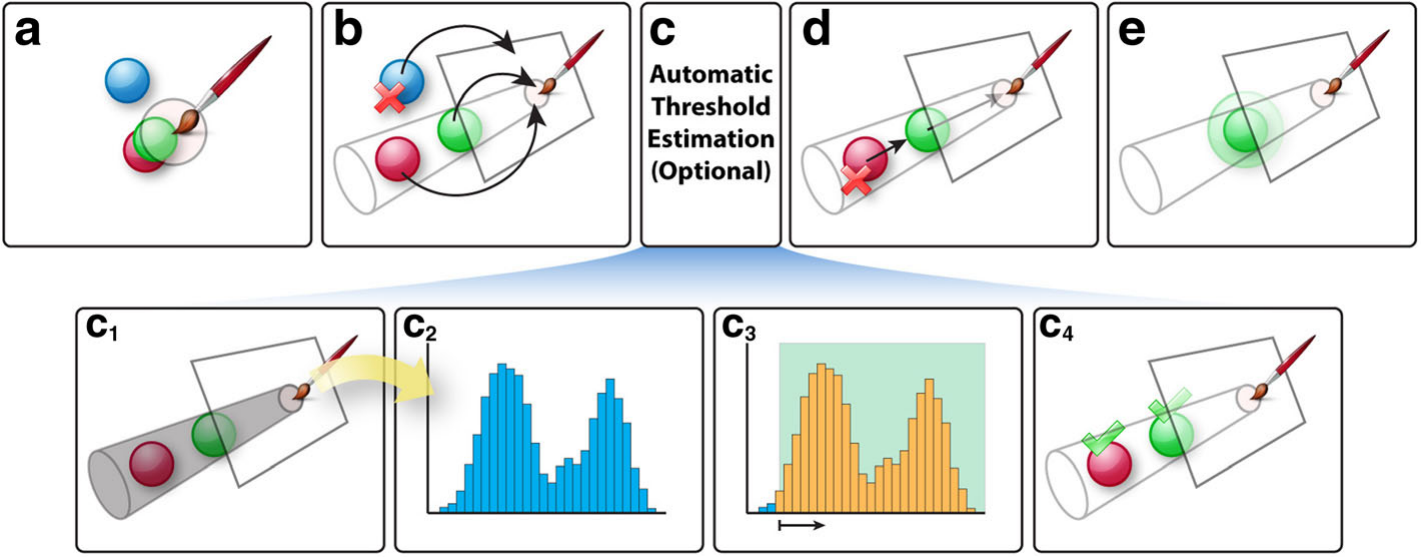
Freehand segmentation. (a) The user uses the mouse to paint on a visualization result to select structures. (b) Voxels are projected. The *blue voxel* is not selected because its projection is outside the painted region. (c) OpenCL kernels are used to estimate a threshold value. (c1) We generate a histogram for voxels within the shaded region. (c2) The histogram from the shaded region. (c3) We calculate a threshold value from the histogram. Low-intensity signals are usually noise and are excluded from the selection. (c4) Voxels with intensities higher than the threshold value are selected. (d) We cast rays backward to the viewer. The *red one* is rejected because its ray is obstructed by the *green object*. (e) We calculate a morphological diffusion to select surrounding structures of the *green object* based on connectivity

FluoRender provides three brush types for mask generation: initial selection, erasing, and fine-tuning of existing selections. All brush operations handle 3D volume data with two familiar segmentation processes in 2D tools: threshold-based seeding and diffusion. To select a structure in 3D, a user first paints with one of the brushes (Fig. 4a). Then, we use a projection lookup to determine whether a 3D sample point falls in the intended selection region. A 3D sample point in a volume data set is projected onto a 2D image space in the visualization process. This projection is performed by a matrix multiplication:

1 $$ p\mathit{\hbox{'}}={M}_{prj}\cdot p $$

In Equation 1, $p^{\prime }$ is the projected point on a 2D image plane, ${M}_{prj}$ is the projection matrix, and $p$ is the data point in 3D. The image plane is eventually mapped to the viewing region on a computer display and visualized. When a user paints with the selection brush in FluoRender, the brush strokes are registered in the 2D image space. Theoretically, we could retrieve the brush-stroke-covered region in 3D by applying the inverse projection matrix ${M}_{prj}^{-1}$. However, the inverse projection is not used, as one point in 2D is associated with an infinite number of points in 3D. In our implementation, we use Equation 1 and uniquely project every voxel of the volume data of a channel to the 2D image space. Then, we check if the projected points fall inside the brush strokes (Fig. 4b). A brush stroke defines two regions in the 2D image space, one for seeds and another for diffusion. Potential seeds as well as the final selection can then be determined. Since the projection is computed independently for each voxel, the computation can be parallelized on GPUs to achieve real-time speed [4].

To easily select and isolate the visualized structures, occlusion between structures in 3D needs to be considered for the brush operations. We use backward ray casting to determine whether a seed point is occluded from the viewer. We calculate a ray emanating from the seed point and traveling back to the viewer (Fig. 4d). Then, we sample along the ray, accumulate intensity values, and check if the accumulated intensity is sufficiently high to occlude the signals behind. The seed is validated when no intensity accumulation is detected, or excluded otherwise [35]. We perform backward ray casting only on potential seed points in parallel, which has a negligible performance impact, as the seed region is usually much smaller than the diffusion region.

Validated seeds are grown to a selection mask by iteratively evaluating their morphological diffusion (Fig. 4e). We designed the morphological diffusion by replacing the gradient terms in an anisotropic heat equation with morphological dilations. An anisotropic heat equation is defined as:

2 $$ \frac{\partial u\left( x, t\right)}{\partial t}=\nabla \cdot \left( g\left( x, t\right)\nabla u\left( x, t\right)\right) $$

In Equation 2, $u\left( x, t\right)$ is the data field being diffused and $g\left( x, t\right)$ is the function to stop diffusion, which is calculated from the boundary information of underlying structures. Heat diffusion usually reaches an equilibrium state (solenoidal field) when divergence of the gradient field becomes zero. When we introduce the morphological terms to replace a standard gradient, the equilibrium state should be a zero-gradient field. Since energy is no longer conserved and divergence-free, the non-zero-gradient field cannot reach an equilibrium state. Therefore, the standard anisotropic heat equation is rewritten as:

3 $$ \frac{\partial u\left( x, t\right)}{\partial t}= g\left( x, t\right)\left|\nabla u\left( x, t\right)\right| $$

Then, we use morphological dilation $\delta \left( x, t\right)$ to evaluate the gradient:

4 $$ \frac{\partial u\left( x, t\right)}{\partial t}= g\left( x, t\right)\left(\delta \left( x, t\right)- u\left( x, t\right)\right) $$

Morphological dilation is defined as:

5 $$ \delta (x)= MAX\left( u\left( x+ b\right)\Big| b\in B\right) $$

In Equation 5, $B$ is a predefined neighborhood of any point $x$ in the field $u(x)$. Finally, we discretize Equation 4 to solve over time steps:

6 $$ {u}_{i+1}(x)={u}_i(x)+ g(x)\left({\delta}_i(x)-{u}_i(x)\right)= g(x){\delta}_i(x)+\left(1- g(x)\right){u}_i(x) $$

The reason to use morphological dilation instead of the standard gradient discretization methods is that Equation 6 can be evaluated very efficiently on GPU. Additional file 1: Supplementary Result 2 compares the execution speeds of several common image- processing filters on the GPU and central processing unit (CPU). In our test, the morphological dilation filter not only consumes less time than most other filters but also achieves the highest speed-up. More importantly, it requires fewer iterations than a standard anisotropic diffusion, as it causes the energy of the field to increase monotonically. Therefore, real-time performance is achieved for freehand selection in FluoRender.

## OpenGL-OpenCL interoperation

Data are immediately shared on the graphics hardware for visualization (using OpenGL) and analysis (using OpenCL) [24, 25]. For low-contrast and noisy data, accurate structure-based selection can be generated by incorporating OpenCL computing kernels into the OpenGL visualization pipeline. For example, a thresholding value for the mask generation process can be manually selected by the user, or automatically computed by refining an existing user selection. The automatic estimation of a proper thresholding value is a typical statistical analysis based on intensity frequency, or histogram [36]. As illustrated in Fig. 4c, when automatic thresholding is enabled and a user performs the paintbrush operation, both the original data and the user-selected 3D mask are processed with an OpenCL kernel to generate a histogram of the original data within the mask (Fig. 4c1-c2). Since these data are shared on the graphics hardware, the OpenCL kernel can leverage parallel computing threads to examine the intensity values of multiple voxels and generate the histogram in real time. The histogram is stored on the graphics hardware as well. A second OpenCL kernel starts processing the histogram once it is generated. We employ the commonly used histogram segmentation method to detect peaks and valleys. We fit the histogram to a standard distribution and choose the thresholding value at the $2\sigma$ ($\sigma$ as the variance) intensity toward the lower end (Fig. 4c3-c4). To an end user, these calculations are transparently executed at real-time speed. A refined 3D mask is generated based on the threshold from the OpenCL kernels. Other analysis functions as well as image processing filters use the same procedure for GPU-based computing.

## Results

In our redesign of FluoRender, each channel is handled as an independent yet interoperable entity and uses streaming to lift the restrictions on the number of channels that can be visualized simultaneously. This unique ideology translates to three distinctive features. 1) The ideology allows an extended number of volume channels to be directly visualized in 3D. 2) It visualizes volume data based on the original intensity values of each channel, without the pseudosurface extraction that often yields a misleading appearance of a structure, and it supports a variety of visualization configurations. 3) Data for visualization and analysis are readily shared on GPUs, ensuring that segmentation and analysis of multichannel data are based on the original intensity values of each channel.

## Multichannel streaming

FluoRender easily allows simultaneous visualization of as many as 96 independent channels (Additional file 2: Video 1), which in previous versions had required conversion to RGB channels or pseudosurfaces. Otherwise, interactive visualization would not have been available. This unique multichannel and real-time rendering capability has successfully assisted detailed visual examination and comparison of a variety of 3D datasets in various biological studies [5–8, 37], featuring from 4–96 channels. The resulting fully volume-rendered images contain richer details and provide more accurate representations of biological structures than previous visualization methods [2, 38–40], which employed surfaces or lines for representing 3D structures.


Additional file 2: Video 1. Visualize the Clonal Units of the *Drosophila* Brain. This video demonstrates visualizing 96 channels of the clonal units in the *Drosophila* brain. All channels can be rendered simultaneously and interactively in FluoRender. One can select a channel by directly clicking in the render view. Then, the settings of the selected channel can be adjusted. A selected channel can also be isolated in the visualization for a focused study. High-resolution video: https://youtu.be/mng0sKqXF6I. (MP4 20321 kb)


## Visualization of channel data

**Fig. 5:**
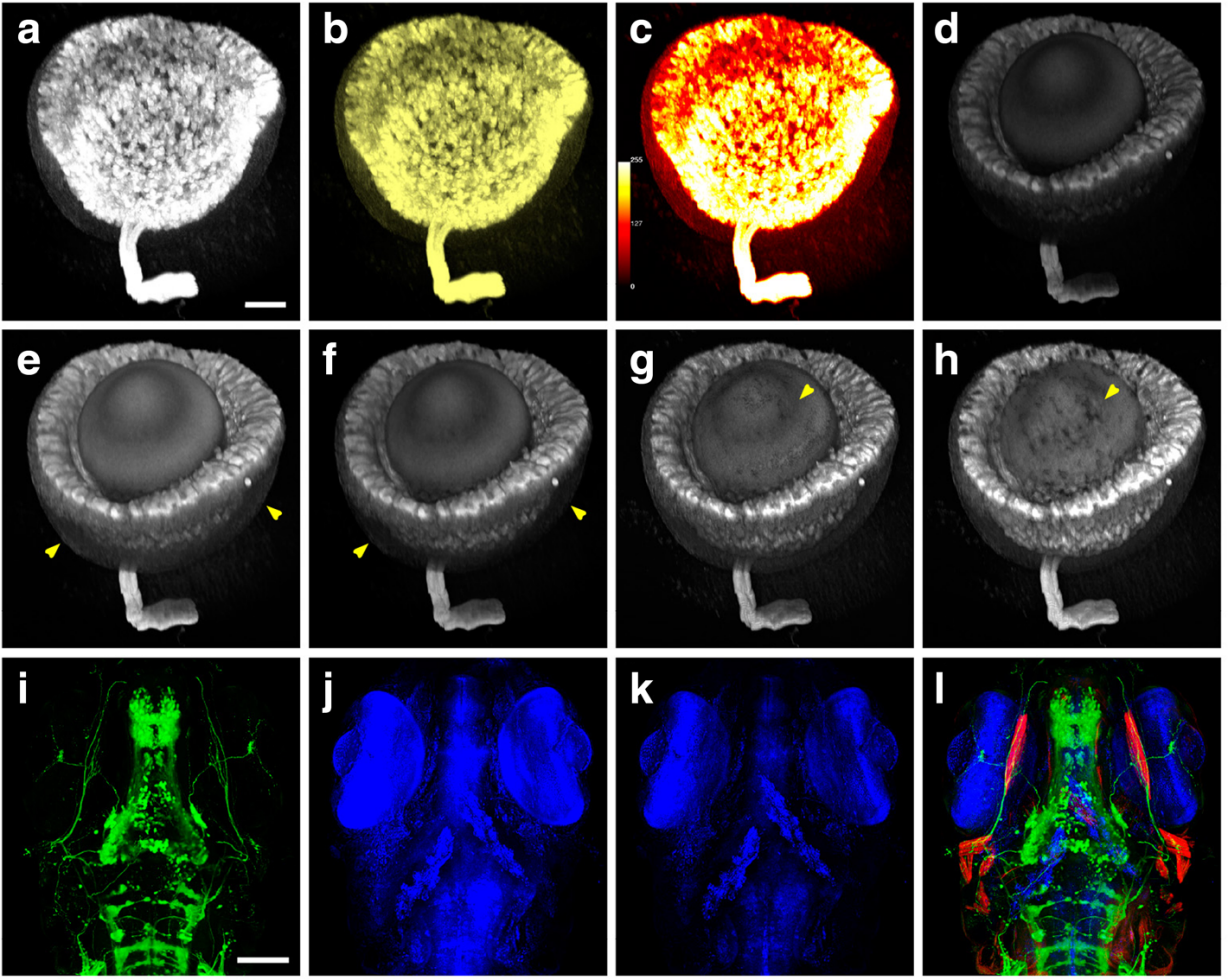
Versatile render modes are available for each volume channel in a multichannel data set. **a** Visualization of a segmented zebrafish eye in MIP mode. Scale bar represents 50 μm. **b** The color of the same volume data is changed to *yellow*. **c** A color map is applied to the data, enabling accurate reading of the intensity values. **d** The same volume data set is visualized in the DVR mode, revealing its actual structure. **e** Gamma is adjusted to reveal areas with relatively low-intensity signals, indicated by arrows. **f** Depth attenuation is adjusted. The areas indicated by arrows are darkened as they are farther back from the viewing point. **g** Shading and shadows are added to enhance textural details. **h** Transparency is increased. The arrow points to an area showing the structures behind the eye ball, which is not seen in **g. i** One channel of the neuronal fibers in the zebrafish embryo is visualized in the DVR mode. The scale bar represents 100 μm. **j** Another channel of the neuronal nuclei is visualized in the MIP mode. **k** MIP and DVR modes are intermixed to enhance depth perception. **l** Three channels of muscles (*red*), neuronal fibers (*green*), and nuclei (*blue*) are intermixed

For a data set containing from a few to over a hundred channels, each channel can be individually adjusted with a series of settings. For example, Fig. 5a visualizes the distribution of the glial fibrillary acidic protein (GFAP) in the developing zebrafish eye with MIP. The flexible assignment of all possible combinations of RGB values permits a rendering of the volume with any desired color, a very important feature when visualizing images that consist of more than three channels (Fig. 5b). Further, assigning a color map enables accurate reading of intensity values (Fig. 5c).

A MIP-based rendering tends to obscure 3D structural information. The DVR method, on the other hand, generates images that reflect the 3D relationship of the structures much more faithfully (Fig. 5d). The Gamma setting controls the brightness of the mid-tones so that structures with intermediate signal intensity can be visualized at a suitable brightness without clipping the bright or dark regions (Fig. 5e). The depth attenuation setting darkens signals farther from the viewing point, providing information about the distance from the observer (Fig. 5f). The addition of shading and shadows further improves the appearance of a channel by providing texture according to the morphology (Fig. 5g). The transparency control provides a solid or translucent representation of the object (Fig. 5h).

## Channel intermixing

To reduce data occlusion in a many-channel data set, the FluoRender visualization pipeline is equipped with a series of modes for intermixing 3D data, which are not available in other tools. For example, the cytoplasmic signal of neurons is best visualized with a translucent DVR rendering for the spatial relationship of the overlapping neuronal fibers (Fig. 5i), whereas the neuronal nuclei are best represented with MIP to detect their presence inside large tissues (Fig. 5j). In addition, more than one rendering method can be combined to visualize the data of a single channel (Fig. 5k). Images of a single channel visualized with such combined rendering modes can be further mixed with those of other channels to present features in an informative way (Fig. 5l, Additional file 3: Video 2).


Additional file 3: Video 2. Study of the Zebrafish Visual System. This video demonstrates using multiple channel render modes to visualize the zebrafish visual system. High-resolution video: https://youtu.be/3q2KGNG5ZVA. (MP4 13904 kb)


**Fig. 6:**
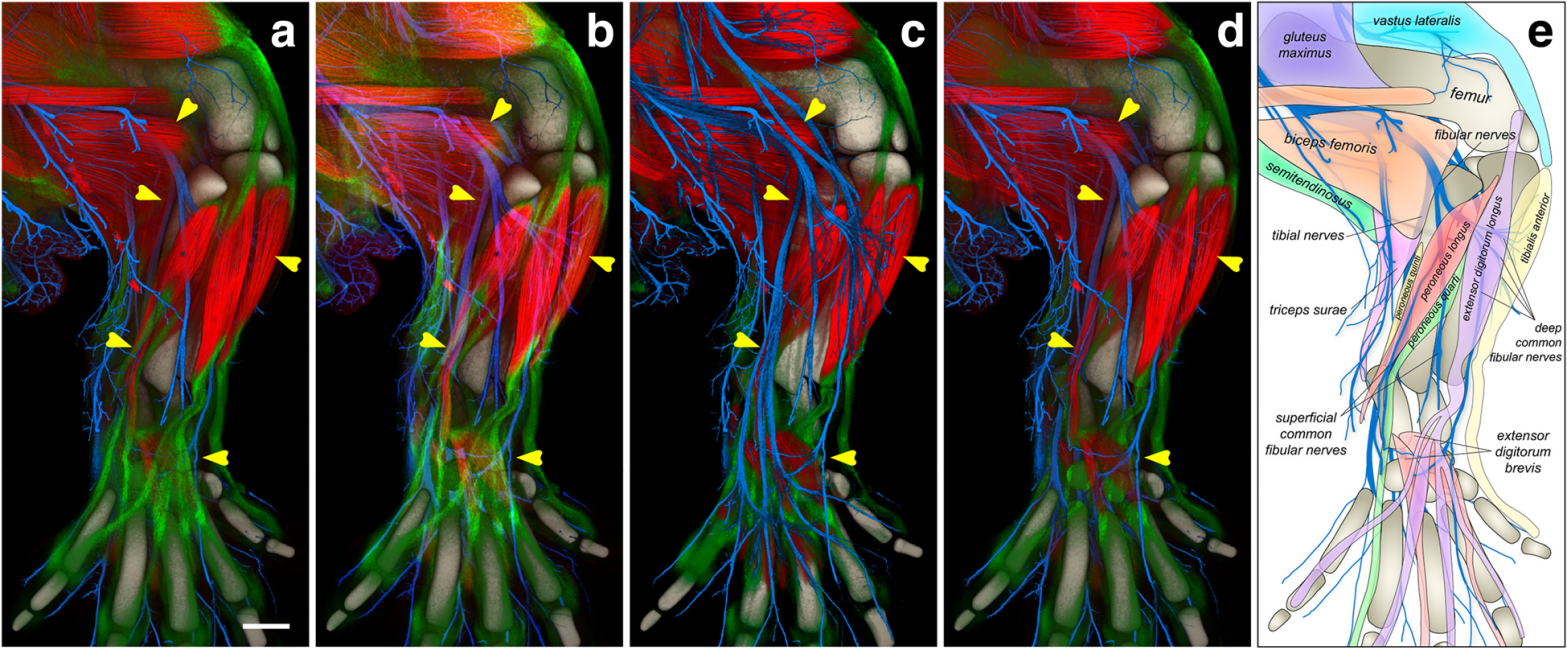
Channel-intermixing modes facilitate the study of the complex anatomy. The confocal microscopy scan of a hind limb of an embryonic mouse contains three channels of muscles (*red*), tendons (*green*), and nerves (*blue*). The bones (*gray*) were extracted from the empty space of the scan. **a** The channels of the scan are intermixed with the depth mode. The fibular and tibial nerves are occluded by a series of muscles and tendons (indicated by the arrows). The scale bar represents 200 μm. **b** In the composite mode, we observe the underlying nerves. However, the spatial relationship between some muscles and nerves is still unclear. For example, it is difficult to tell if the deep fibular nerves innervate the extensor digitorum brevis muscles. **c** The layered mode visualizes the nerves, muscles, tendons, and bones from top to bottom. The structures of the nerves are most obvious. **d** The layered mode groups the muscles and nerves, which are above the tendons. The group of muscles and nerves is rendered with the depth mode. We observe that a lateral branch of the superficial fibular nerves innervates between the peroneal muscles following the muscle fiber directions; the deep fibular nerve innervates the extensor digitorum brevis muscles at an angle to the muscle fiber directions. **e** Our knowledge obtained from a combination of different channel-intermixing modes is illustrated in a cartoon, clearly showing the anatomy

A choice of channel intermixing modes as well as their combinations using groups allows easy adjustments for emphasizing different structures. For example, Fig. 6a shows a 3D visualization of the developing hind limb of a mouse embryo, in which muscles, tendons, nerves, and bones are visualized in separate channels. In this visualization mode, the occlusion of biological structures among different channels is visually correct: dense volume visualized in one channel occludes not only the background structures of the same channel, but also those visualized in other channels. Although the depth mode provides the visually correct spatial relationship, complex structures in deep regions tend to be occluded by superficial ones, especially when the number of channels increases. It becomes difficult to understand the full structure visualized in a specific channel. The composite mode addresses this problem by accumulating instead of occluding the signals of individually rendered channels (Fig. 6b). The intensity values of all channels can be recognized at colocalized sites; deep objects visualized in one channel can be seen through structures of other channels in front. The accumulation of multiple channels affects the appearance of the colors, making it sometimes difficult to trace the structure visualized in a specific channel. In biological visualization, it is a common practice that information in one or two channels is prioritized over the others, which represent “background” labeling just for showing the overall morphology of a sample. Rendered images of the background channels should not obstruct those of higher importance. The layered mode is designed to satisfy such needs by superimposing individually rendered channels on top of one another (Fig. 6c). Using this mode, channels of higher importance (the nerves) are visualized in the foreground for close inspection, whereas other channels are used as a reference in the background.

However, several channels are equally important. A combination of different channel-intermixing modes is needed. In Fig. 6d, the channels representing nerves and muscles are grouped with the depth mode and placed with the layered mode so that they appear in front of the other two channels (tendons and bones), which are also grouped with the depth mode. The complex spatial relationships between nerves and muscles are visualized because they are not obscured by the signals of tendons and bones. Different channel-intermixing modes can be interactively switched for a better understanding of the spatial relationship of different anatomical features (Fig. 6e, Additional file 4: Video 3).

## Floating-point rendering

**Fig. 7:**
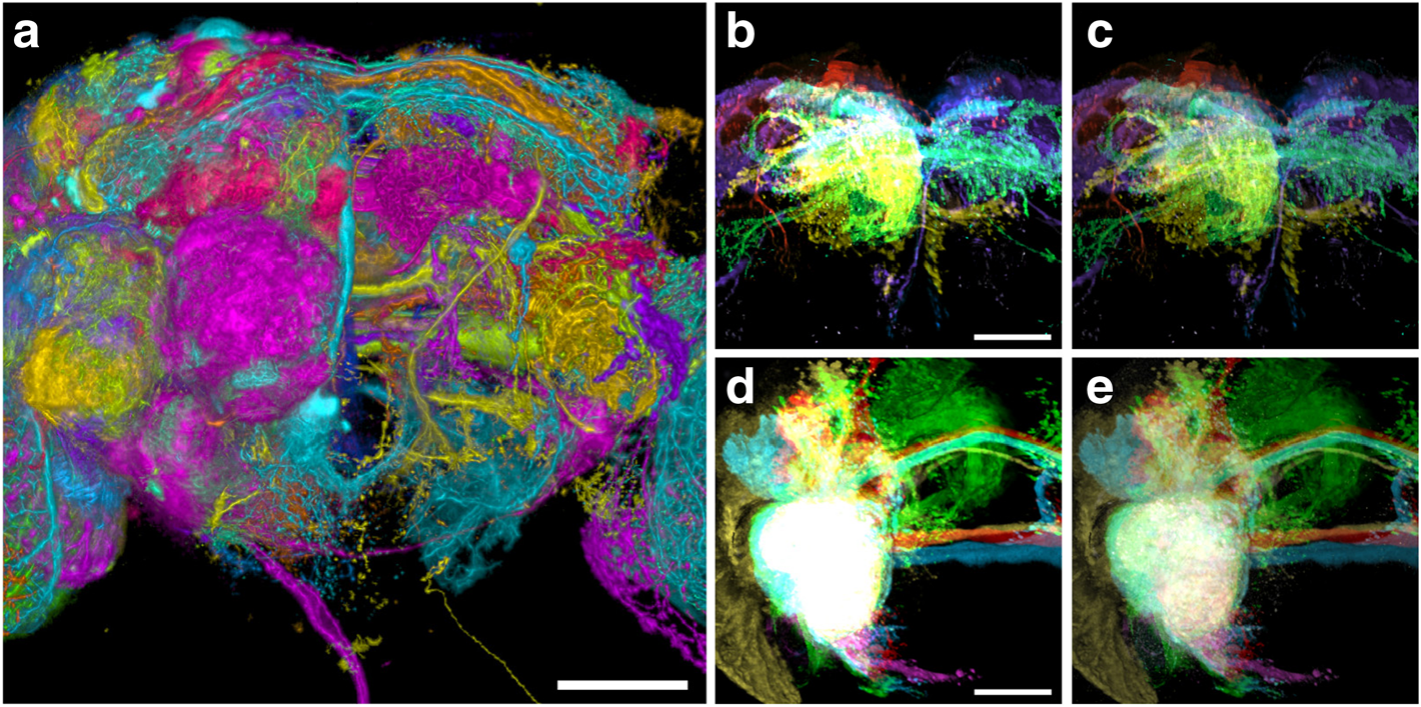
Tone-mapping adjustment and versatile render modes facilitate multichannel intermixing. **a** Visualization of all 96 identified clonal units of the *Drosophila* brain, anterior view. The scale bar represents 50 μm. **b** Rendering of seven clonal units of the superior medial protocerebrum (SMP) in the composite mode. Without the tone-mapping adjustment, the details of high-intensity values are lost. The scale bar represents 50 μm. **c** The same clonal units in b rendered with the tone-mapping adjustment. Arborization patterns of different channels are observed. **d** Close view of five clonal units of ventrolateral protocerebrum (VLP), without the tone-mapping adjustment. The entire region of overlapped arborization patterns is clipped to a *white color*. The scale bar represents 50 μm. **e** The same clonal units in d rendered with the tone-mapping adjustment


Figure 7 demonstrates the advantage of FluoRender’s HDRI feature. Figure 7a shows the visualization of almost all neural projections in the brain of an adult fruit fly *Drosophila*. Each channel, in a different color, shows an individual clonal unit – a lineage-defined group of projections of the neurons deriving from each neural stem cell [1, 5]. Those data are then integrated into a single multichannel view. The channels are grouped in a hierarchical manner to enable selective visualization (Additional file 2: Video 1). The depth-mode rendering shown here faithfully represents the spatial relationship of all clonal units (Fig. 7a). However, neurons in deep brain regions are obscured by those in the foreground. Fig. 7b and d show selective clonal units that arborize in specific brain regions. The composite mode is employed here so that overlapping signals can be visualized without occlusion. However, even though only a handful of channels (seven and five, respectively) are visualized in these images, extensive overlap of the visualized channels results in the accumulation of very high-intensity values in many areas, causing signal clipping and detail loss. Such detail can be preserved when we apply the interactive tone mapping (Fig. 7c and e, Additional file 5: Video 4).

Additional file 5: Video 4. Study of the Clonal Units of the *Drosophila* Brain. This video demonstrates using tone mapping to recover the saturated signals when multiple channels are visualized together. Colocalized structures and details can then be qualitatively studied. High-resolution video: https://youtu.be/7G4i_lIyCvw. (MP4 12654 kb)


## Freehand segmentation

**Fig. 8:**
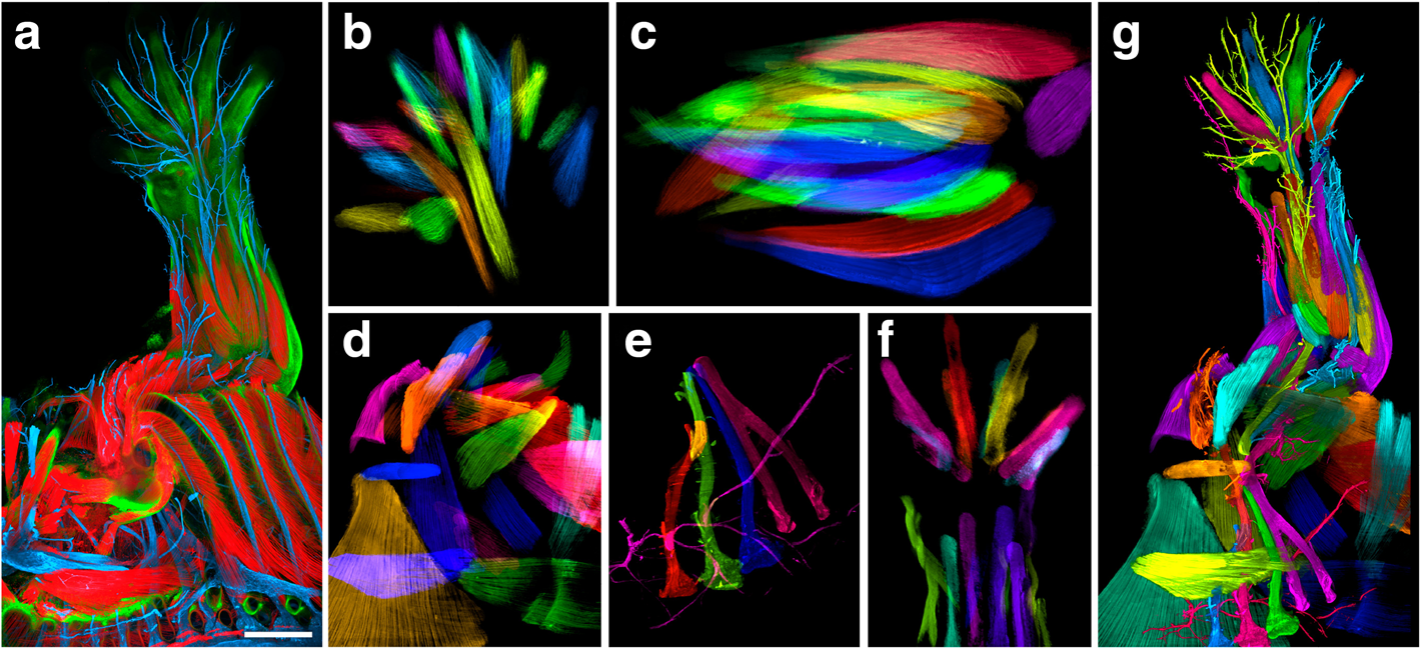
Making and visualizing a mouse forelimb atlas from confocal microscopy scans. **a** The original scan contains three channels of muscles (*red*), tendons (*green*), and nerves (*blue*). The scale bar represents 500 μm. **b** Using brush-based SOI selection and extraction, we segment 21 muscles of the paw. Each muscle is an independent channel with adjustable settings. **c** The 22 segmented muscles of the forearm. **d** The 22 segmented muscles around the shoulder area. **e** The 5 segmented spinal nerves going down to the forelimb. **f** The 17 segmented tendons of the forearm muscles. **g** We segment the anatomical structures of the forelimb into 91 channels. Each channel is assigned a color for visualization

A selected structure can be extracted from the original channel to become a new channel. A multichannel data set of individually extracted structures can be visualized and analyzed using FluoRender. This method was used to generate the *Drosophila* brain atlas in Fig. 7, where each channel was extracted from an individual scan. Additionally, the method was used for making an atlas of the embryonic mouse limbs to study normal anatomy as well as identify congenital abnormalities. The original 3D volume data of a mouse forelimb (Fig. 8a) consisted of three channels for muscles (red), tendons (green), and nerves (blue). From these data, individual muscles, tendons, and nerves were segmented into 91 channels to visualize with different colors and transparency values and to remove irrelevant structures for the final image (Fig. 8b-g).

## OpenGL-OpenCL interoperation

**Fig. 9:**
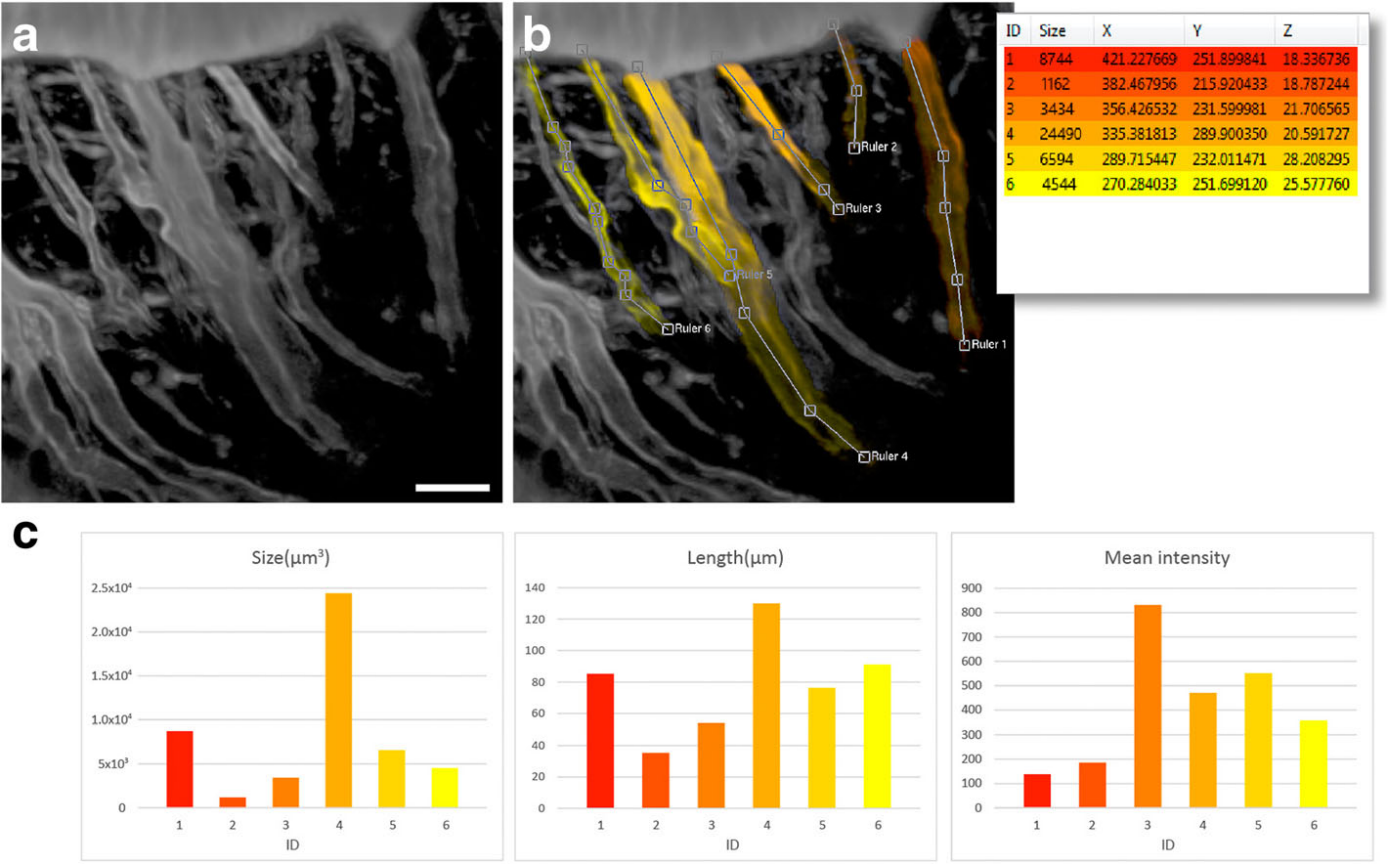
Analysis of six nerves from one channel of a confocal scan. The scan is an inner ear of the vestibular system (*Opsanus tau*). **a** Volume rendering of the scan. The nerves are different in size, length, and intensity. The scale bar represents 20 μm. **b** We select and define six SOIs, each representing a nerve. We also use a ruler tool to measure the length of each selection. The inset shows a list of the selected nerves, which are color-coded according to their IDs (1–6). **c** The selected nerves are differentiated and compared by performing a series of analysis functions, including calculations of volumetric size, length, and mean intensity. The results are shown in the charts. The intensity range of the scan is 0-4095 for a 12-bit microscopy file format


Once an SOI is segmented, a variety of quantitative analysis can be performed. Built-in analysis functions include connected component analysis, size analysis, intensity analysis, etc. Intuitive measurement tools for position, length, angle, size, etc. can be directly applied to volume channels, where anchor points are placed three-dimensionally based on intensity values. OpenCL kernels are seamlessly integrated with OpenGL rendering procedures to perform these analysis tasks. Sharing data between OpenGL for visualization and OpenCL for computing further enables real-time processing and analysis. For example, about an order of magnitude of speed-up (performance increase measured by decrease of computing time [41]) can be achieved for common image processing filters [36] (Additional file 1: Supplementary Result 2 and Additional file 6: Video 5), which makes interactive data processing much more efficient, giving users the opportunity to achieve the most informative data extraction within the same period of analysis work. Figure 9 demonstrates the analysis of selected nerves in an intact ear semicircular canal of an oyster toadfish (*Opsanus tau*) (also Additional file 7: Video 6).

Additional file 6: Video 5. OpenCL Filters in FluoRender. This video demonstrates using OpenCL for image processing. Commonly used 3D filters can be programmed within FluoRender’s user interface similar to a script language. The execution of these filters on GPU is generally several times faster than their CPU implementations. High-resolution video: https://youtu.be/okp_zijKu5U. (MP4 16516 kb)



Additional file 7: Video 6. Analysis Functions of FluoRender. This video demonstrates the analysis functions in FluoRender. A paintbrush tool can be used to select desired structures in 3D. The selections can have their length and signal intensity measured. A comparison between volume-based analysis and polygon models also demonstrates the accuracy and reliability of the former method. High-resolution video: https://youtu.be/CV3iCZMO8C4. (MP4 19560 kb)


## Comparing FluoRender to similar tools

We surveyed the multichannel capabilities of the commonly used software packages in biomedical research, detailed in the Additional file 1: Supplementary methods and results. Table 2 summarizes the key features of FluoRender when comparing it to similar tools in the survey. FluoRender is currently the only tool capable of many-channel volume data processing and visualization, using a workflow entirely based on volume intensity values without pseudosurface conversion or channel combination.

**Table 2:** A comparison of multichannel processing and visualization features in popular tools

	Amira	Imaris	Vaa3D	Volocity	FluoRender
Open Source (Free)	No	No	Yes	No	Yes
Channel Number (Tested)	RGBA	25	RGBA	16^a^	96^b^
Time to Load Data^c^	20 min	5 min (For 25 channels)	2 min (w/ Renaming)/10 min	1 min	1 min
Channel Adjustment	Not interactive	Interactive	Not Interactive	Not always interactive	Interactive
Channel Intermixing	Depth	Depth	Depth	Depth	Multiple
Floating-point/Tone Mapping	No	No	No	No	Yes
Volume Selection/Extraction	No	No	No	No	Yes

^a^Volocity may have corrupted rendering results depending on the number of channels

^b^The test data set contains 96 channels. More can be supported by design

^c^Time for an experienced user when manual work is required. An ordinary hard drive disk was used (4 TB/7200 RPM). Imaris was not able to load more than 25 channels. Therefore, the loading time was measured for 25 channels

## Discussion

In the biomedical sciences, many original findings are the result of observations. Visualization-based analysis is gaining popularity as data complexity increases. We have redesigned the FluoRender tool to address a special demand of interactively visualizing and processing complex volume data sets consisting of many channels. Applications in biomedical research that benefit from our tool include segmentation, comparison, analysis of many channels of volume data from mutant-control scans, and the construction of anatomical atlases. The lack of proper multichannel support in previous tools has forced researchers to choose inaccurate data representations, such as pseudosurfaces, which are inadequate for visualization and analysis of grayscale values. Similar to the previous pseudosurface-based representations, interactivity is crucial for users to understand a complex data composition with a large number of volume channels. To maintain the interactivity of FluoRender and to visualize a greatly expanded number of channels, we restructured its rendering pipeline and designed multilevel data streaming as well as triple-buffer compositing. We demonstrated that volume-based atlases containing about 100 channels were interactively visualized using personal desktop computers. Our design supports more channels with the same flexibility. The conjoining of accurate details and the capability of interactive exploration would otherwise not have been possible.

Furthermore, the significance of our methods, which maintain each grayscale channel in a multichannel volume data set as an independent entity, is the flexibility for GPU-based processing and analysis. Avoiding pseudosurface or RGB conversion, our method loads original grayscale values into graphics hardware for both rendering and computing at interactive speed. Therefore, we are able to provide versatile visualization and analysis features, most of which are unavailable elsewhere. Each channel can have its own render modes and settings, thus providing a wide range of choices for maximizing the clarity of the visualized results. Channels can be intermixed with different modes, so that features from specific channels can be easily accentuated, instead of being occluded. A 3D mask can be associated with one channel, allowing SOI-based data processing and analysis, including extraction, connected component analysis, and statistical analysis. Additionally, we designed an intuitive method to select SOIs by painting directly on the visualization results, a familiar operation adopted by popular 2D imaging packages such as Photoshop [42].

An arbitrary interaction speed can be set on any computer system using the streaming method. However, the amount of data that can be processed and rendered within one streaming loop is limited by a system’s computational capacity. Artifacts such as flickering can be observed on computers not equipped with high-end graphics cards. Preprocessing and building a pyramidal data structure and selectively rendering lower levels of detail based on interaction speed are common methods for visualizing large data. Flickering can thus be replaced with data blurring, which is visually less distractive. Our current many-channel visualization pipeline has not supported the pyramidal data structure because of two considerations. 1) The many-channel data sets we worked with can contain several hundred channels, but each channel is relatively small (up to 1000^3^). Building a hierarchy of such spatial resolutions can produce extremely blurry visualization results. 2) More importantly, our design needs to support interactive processing of a many-channel data set, such as segmentation by painting. Unlike rendering data exclusively, the operations for interactive processing cannot be performed *a priori*. A simple propagation of data processing results among spatial hierarchical levels burdens the system, producing a less interactive experience. Therefore, we maintained interactivity and functionality for many-channel data, with a sacrifice of quality on low-specification systems. Our implementation also limits the data size of one channel, which usually needs to fit in graphics memory. Nevertheless, in future work, we plan to explore postprocessing methods for hierarchical data, so that data processing results can also be interactively propagated among spatial hierarchical levels.

## Conclusions

With a steady increase in computational power and the memory capacity of consumer graphics hardware, it is unlikely that the applications of many-channel volume visualization and analysis will be limited to building and analyzing anatomical atlases. Multispectral scans from microspectroscopy contain more than just RGB channels and naturally fit into our workflow; simulations of biochemical reactions within living organisms generate multivariable data, which can be visualized and analyzed as multichannel; and research on multimodal imaging, which seeks the advantages of different imaging techniques for a data amalgamation, also requires a multichannel workflow. We have demonstrated that a truly volume-based multichannel workflow enables the versatility of adjusting, intermixing, measuring, and editing data, all with intuitive operations suitable for generalized analysis. Our implementation, FluoRender, is distributed under an open-source license. Precompiled executables are also available for both Windows and Mac OS X at www.fluorender.org. The extended multichannel capacity of FluoRender also laid the groundwork for future development. Specifically, we will enable interactive multichannel comparison and organization for visualizing and correcting structural discrepancies/alignment errors in atlas data, multichannel colocalization analysis, and control-mutant comparisons.

## Additional files

Additional file 1: The supplementary material includes supplementary methods and supplementary results. (DOCX 29 kb)

Additional file 4: Video 3. Study of the Mouse Limb Anatomy. This video demonstrates visualizing an anatomical atlas of an embryonic mouse hind limb. Different channel intermixing modes are used to reveal occluded 3D structures. High-resolution video: https://youtu.be/fF5fIVQzyvQ. (MP4 19482 kb)

## Abbreviations

3D: three-dimensional
API: application programming interface
CPU: central processing unit
CT: computed tomography
DVR: direct volume rendering
GFAP: glial fibrillary acidic protein
GPU: graphics processing unit
HDRI: high dynamic range image
MIP: maximum intensity projection
OpenCL: open computing library
OpenGL: open graphics library
RGB: red-green-blue
ROI: region of interest
SOI: structure of interest
VTK: Visualization Toolkit
