# Supplementary material for: FluoRender: joint freehand segmentation and visualization for many-channel fluorescence data analysis
Source: BMC Bioinformatics. 2017 May 26;18:280. doi: 10.1186/s12859-017-1694-9 (PMC5446689; doi:10.1186/s12859-017-1694-9)
Supplement: Supplementary file 6 — The supplementary material includes supplementary methods and supplementary results. (DOCX 29 kb) [file 12859_2017_1694_MOESM1_ESM.docx]

FluoRender: joint freehand segmentation and visualization for many-channel fluorescence data analysis

Supplementary Material

Yong Wan ^1^, Hideo Otsuna ^2^, Holly A. Holman ^3^, Brig Bagley ^1^, Masayoshi Ito ^4^, A. Kelsey Lewis ^5^, Mary Colasanto ^6^, Gabrielle Kardon ^6^, Kei Ito ^4^, Charles Hansen ^1^

^1^ Scientific Computing and Imaging Institute, University of Utah, USA

^2^ Janelia Farm Research Campus, Howard Hughes Medical Institute, USA

^3^ Department of Bioengineering, University of Utah, USA

^4^ Institute of Molecular and Cellular Biosciences, University of Tokyo, Japan

^5^ Department of Biology, University of Florida, USA

^6^ Department of Human Genetics, University of Utah, USA

Corresponding author: Yong Wan (wanyong@cs.utah.edu)

# **Supplementary Methods**

## ***Drosophila* Brain Atlas**

For the clonal units of the *Drosophila* brain in Figure 3, a total number of about 5000 *Drosophila* brain samples were imaged and analyzed. For each sample, one neuroblast was randomly tagged with fluorescent proteins at the beginning of the larva stage, the projection pattern of whose progeny was then analyzed in the adult. Ninety-six clonal units in the cerebrum were thus identified, including eighty groups of clonal units with a single cluster of cell bodies, fourteen groups of cells featuring two or three clusters, and two potential clones. The neural projections of the clones were artificially colored within FluoRender to distinguish each one. For detailed information on generating the clonal unit atlas of the *Drosophila* brain, please refer to the paper by Ito et al. (<http://dx.doi.org/10.1016/j.cub.2013.03.015>).

## **Mouse Embryonic Limbs**

ScxGFP mouse limbs were provided by Ronen Schweitzer (http://dx.doi.org/10.1002/dvdy.21179) and labeled via wholemount immune-fluorescence. Muscles were labeled with My32 antibody to neonatal, nerves were labeled by antibody to neurofilament, and tendons were labeled by antibody to GFP (labeled via Scx) according Merrell et al. (http://dx.doi.org/10.1038/ng.3250). Mouse limbs were cleared in benzyl alcohol:benzyl benzoate (1:2) and scanned using Nikon A1 confocal with a 10x objective. Mice were housed in an AALAC-approved animal facility and embryonic mice were harvested and sacrificed via CO_2_ inhalation at defined time points. The experiments on mouse embryonic limbs were approved by the University of Utah Institutional Animal Care and Use Committee (IACUC 16-06007).

# **Supplementary Result 1. A Survey of the Multichannel Capabilities of Commonly Used Tools in Biology**

We surveyed the multichannel capabilities of the commonly used software packages in biomedical research and report our results here. Notice that the meaning of the term “multichannel” may vary for different tools. An RGB volume, which is often termed a multichannel data set, is in fact stored as one data object in OpenGL. Some tools consider pseudosurfaces (polygonal models) as channels. Here, the multichannel capability specifically refers to the software features for visualizing and processing an indefinite number of volume channels. Packages lacking the capability of visualizing more than red, green, and blue channels are therefore disregarded. For example, a large number of channels can be stored with ImageJ’s hyperstack feature, but not visualized altogether three-dimensionally. In the following sections, four tools are examined: Amira, Imaris, Vaa3D, and Volocity. We used the 96-channel *Drosophila* brain atlas for all the tools we tested. For each tool, we first present the phenomenon when we were trying to load as many channels as we could from the test data set. The user experience of the tool is quantified as the time spent for the operations. Then, we discuss the tool’s implementation that produced the observed results. Both advantages and disadvantages of its implementation are listed next. Finally, we discuss its typical use and potential improvements.

All tests were performed on a Windows desktop computer with an Intel Core i7 3.4GHz CPU (Haswell-E), 32 GB DDR4 system memory, and an Nvidia Quadro M6000 (GM200) graphics card with 12 GB GDDR5 graphics memory.

## **Amira, Version 5.6**

**Phenomenon:**

The multichannel visualization was achieved with the “MultiChannelField” object. (A data processing module in Amira is usually termed an object, which can be connected with other objects to form a data processing pipeline.) Objects containing the volume channels of the test data set were connected to the MultiChannelField object. Then, the MultiChannelField object was visualized using a volume rendering object, “Voltex”. All channels could be loaded into Amira. Initially, Amira assigned a different color to each channel. The color of a channel was the only adjustable parameter. Whenever the color of a channel was changed, an “Apply” button had to be clicked in order for the change to take effect. Then, it took several seconds for the result to be updated. The update took more time when the number of channels increased. There were no interactions with the multichannel visualization, such as selecting and extracting grayscale values directly from the view.

**Time consumed:**

About 20 minutes for loading channels, since a significant amount of manual work was involved. Adjusting the colors took about another 20 minutes.

**Implementation:**

The MultiChannelField object combines all input volume channels into an RGB volume in preprocessing. The combined volume is then rendered as a single data set. Each time the “Apply” button is clicked, the channel combination is recalculated.

**Advantages:**

1. The combined volume is visualized with interactive speed.

2. There is no practical limit to the number of channels that can be visualized.

**Disadvantages:**

1. The object-connecting operations for all 96 channels is time-consuming. A series of repetitive operations has to be performed for each channel.

2. Very few adjustments can be made to the visualization result.

3. The overall visualization experience is noninteractive because parameter adjustments take a significant amount of time.

4. There is occlusion between channels because of the lack of versatile channel intermixing modes.

5. No further interactive processing and analysis can be made to a visualized volume, because original data values are lost in the channel combination process.

**Comments:**

If data presentation is the sole purpose, Amira can be used for visualizing a large number of volume channels. However, the process of adjusting for good visualizations can be time-consuming. Amira is typically used to convert volume channels and segmented structures into pseudosurfaces and then visualize the converted data. Since Amira can be expanded by the introduction of new objects, an updated multichannel model can be implemented in place of the MultiChannelField object. Instead of combining channels in preprocessing, the new module will intermix channels in postprocessing. There is also the potential of adding interactive processing and analysis tools into Amira as new modules, allowing, for example, 3D ruler tools and direct channel selection.

## **Imaris, Version 7.7**

**Phenomenon:**

Imaris allowed loading and visualizing just one channel initially. Then, an “Add Channel” command was used to add more channels to the visualization. However, when the number of channels reached 25, Imaris reported insufficient texture memory and refused to load more. The parameters of successfully loaded channels could be adjusted in the “Display Adjustment” panel. The parameters included a color map, min/max values, Gamma, and opacity. The view port update after a parameter change was interactive. Individual channels could not be selected directly from the visualization. A 3D ruler tool measured lengths in 3D. However, the depth calculation of the 3D ruler tool relied on the maximum intensity value, lacking an option to place ruler points on the structure exterior (whose calculation relies on the accumulated intensity of ray casting). There was no tool to directly select and extract grayscale values from the visualizations.

**Time consumed:**

About 5 minutes to load channels until no more channels could be added.

**Implementation:**

Among the tested tools, the multichannel visualization of Imaris was generally interactive. Imaris stores data from individual channels in the graphics memory. However, it combines these channels in an OpenGL rendering shader program. It binds all loaded channels to the texture-mapping units of the GPU and calculates the color combination for each sample point within the shader. A limit can be easily reached because of insufficient texture mapping units, insufficient texture memory, or increased complexity of the shader code (refer to developer.apple.com/opengl/capabilities/index.html and feedback.wildfire-games.com/report/opengl for lists of OpenGL capabilities on various graphics hardware and operating systems). Therefore, 25 of 96 channels were visualized.

**Advantages:**

1. For a relatively small number of channels, the visualization is interactive.

2. The rendering parameters can be adjusted interactively.

**Disadvantages:**

1. A limit to the number of visualized channels can be easily reached.

2. Only a MIP mode is available to reduce channel occlusion. However, the MIP mode obscures 3D structures.

3. There are no tone-mapping operations to recover the clipped signals when the MIP mode is used.

4. Since all texture-mapping units have been allocated for rendering volume channels, none are left for 3D mask volumes. Therefore, it becomes difficult to add direct grayscale value selection and extraction. Visualizing the results from mask-selected volume channels would also be difficult.

**Comments:**

If the number of channels is less than 20, Imaris can be useful for generating satisfactory visualizations. In order to support more channels, what is lacking in Imaris is data streaming. If channels were to be rendered and intermixed sequentially, only one texture mapping unit would be sufficient at one time for rendering. The extra texture- mapping units can be used for mask volumes and identifier volumes (for segmenting multiple structures from one channel). It not only can solve the issue of a limited number of channels, but also allow many interactive processing and analysis functions to be performed on GPUs. In addition, the code for visualization and processing becomes less complex and more maintainable. As a side note, in our tests of Imaris, we noticed that it used precalculated, axis-aligned slices for volume rendering. Although it achieved a fast rendering speed, artifacts could be easily observed, especially when one set of slices was switched to another during view rotation. Using view-aligned slices for volume rendering should significantly improve Imaris’s volume rendering quality.

## **Vaa3D, Version 2.9**

**Phenomenon:**

Vaa3D allowed opening one channel at a time, each channel being visualized in its own window. A tool in Vaa3D, called “image blending”, could be used to generate a combined channel from the opened data. There was also a dialog window similar to an installation wizard, guiding users through the channel-blending configurations. For each channel to be blended, one was asked to choose its name, channel index, and weights for red, green, and blue colors. However, this process became impractical for more than 10 channels, as it was difficult to keep track of all the channel names and their color weights, which had to be repeatedly filled in the wizard dialog. Alternatively, one could rename the files of the test data set to a common identifier plus index numbers. Then, all the channels could be imported with randomly assigned colors. The second method was easier to use for a large number of channels, but still difficult for a user to keep track of all channels, since their original names were changed. Eventually, a blended RGB volume was generated for 3D visualization by either method. Remapping the intensity or transparency of the newly generated red, green, and blue channels did not work for our test data set, since red, green, and blue channels had lost their biological meanings after channel combination. Vaa3D allowed managing and visualizing the multichannel data in 2D. Weights of each channel could be changed. For 3D, Vaa3D provided a tool, called Virtual Finger, which allowed direct extraction of structural skeletons from volume data. However, it could not be applied on the blended volume, since the calculation was based on the combination of red, green, and blue colors, instead of the values from the original channels. Therefore, its use for multichannel data was limited in 3D because of the channel-blending process.

**Time consumed:**

About 2 minutes to load channels when the files had been renamed. Otherwise, it took more than 10 minutes to load each channel. To combine channels manually, it took about half an hour, although it is recommended to plan the channel colors in a spreadsheet beforehand, the time of which is not included.

**Implementation:**

Similar to Amira, Vaa3D preprocesses and combines multiple channels into one RGB volume. The RGB volume is then treated as one original data set, which can be visualized and analyzed using its existing toolset.

**Advantages:**

1. The combined volume is visualized with interactive speed.

2. There is no practical limit to the number of channels that can be visualized.

3. Vaa3D provides a useful multichannel management system in 2D.

**Disadvantages:**

1. The channel-blending operations for more than 10 channels can be confusing.

2. Signals may be clipped after channel blending, since only 8-bit textures are used.

3. Channel adjustments for 2D and 3D are separate because the blending process generates different data for 3D.

4. Channel occlusion could not be solved in 3D due to the lack of channel intermixing modes.

**Comments:**

Vaa3D is useful for single and RGB channel data visualization and analysis. It can also be used for more channels when visualization is in 2D. Its channel blending is difficult to use for a large number of channels. Many of its 3D tools, such as the Virtual Finger, are based on RGB data. Since RGB channels are the basic data model for Vaa3D in 3D, it lacks the flexibility to adapt to multispectral data with an indefinite number of channels. However, since Vaa3D uses GPUs to visualize RGB data, it has the potential of incorporating more interactive tools such as the Virtual Finger, as well as taking advantage of the GPU computing for a more interactive processing speed.

## **Volocity, Version 6.3**

**Phenomenon:**

All the channels of the test data set could first be loaded and organized into a library. Each channel was in its separate folder as a sequence of image sections. Then, one could select multiple channels in the library and right-click the selection to show a context menu. A command, “Create new image sequence from selection”, could be used to create a new image sequence containing multiple channels. However, when all 96 channels of the test data set were added to the new image sequence, no visualization result could be obtained. Therefore, an attempt was made to reduce the number of channels by turning off the display of each channel. This was a slow process, as Volocity became unresponsive for several seconds when a channel was turned off. It was found that Volocity’s standard volume visualization method, “3D opacity”, could support no more than 16 channels. When the displayed channels were of certain numbers, despite being less than 16, the visualization from one channel might become corrupted. It was also found that its “3D ray tracer” method could support more channels. However, it took more than 10 minutes to generate a still rendering of 10 channels, which could not be used for any interactive visualizations. Visualizing more channels using the ray tracer turned out to be impractical. When the “3D opacity” method was used and the number of channels was fewer than 10, the visualization was interactive. Depending on the specific parameter being changed, some parameters were applied in real-time, whereas some needed several seconds to update. For example, visualization results of adjusting brightness and density were updated in real-time; hiding or showing a channel required several seconds to update. Furthermore, channels could not be directly selected from the visualization, and there were no interactive tools such as grayscale value selection and extraction in Volocity.

**Time consumed:**

It took about 1 minute to load all channels. Since the 96 channels could not be visualized in 3D, it took about 10 minutes to turn off the channels until 16 were shown. If only 16 channels were loaded, it took less than 1 minute.

**Implementation:**

Volocity packs the original channels into RGB volumes, so that each original channel occupies one color channel in an RGB volume. More RGB volumes are created when more than three channels are packed. When one channel is added to or hidden from the visualization, the packing is recalculated. Many operations in Volocity, including channel packing, are performed on the CPU. Since Volocity does not cache channel data in the system memory, it reads data from the hard drive each time data configurations are changed. This causes the system to become unresponsive when, for example, the display of a channel is turned on/off. The channel packing also makes the management of data from multiple channels a complex task. A careless implementation can lead to a channel becoming corrupted in certain situations, which is further responsible for the limited number (16) of channels that can be interactively managed and visualized.

**Advantages:**

1. Volocity works well when there are three or fewer channels.

2. In ray tracing mode, it relies on the CPU to generate a high-quality still image, which theoretically can support a large number of channels.

**Disadvantages:**

1. The number of channels that can be displayed interactively is limited.

2. Changing certain parameters of channels can make the system unresponsive.

3. Packing channels into a series of RGB volumes makes Volocity’s implementation complex. Future extensions, especially interactive functions that use GPU, can also be difficult to implement.

4. There is occlusion among channels because of the lack of channel intermixing modes.

**Comments:**

We feel the implementation of Volocity’s interactive volume visualization is outdated; it cannot take advantage of modern computer hardware. The interactivity of Volocity could have been improved at several places if the system memory and GPU had been better utilized. The CPU-generated visualizations are of high quality. However, parameters for such visualizations are difficult to adjust, due to the lack of reliable interactive options.

## **Conclusion**

Visualization and analysis of multichannel volume data from fluorescence microscopy have not been the strengths of the commonly used software packages for biomedical research. When more than red, green, and blue channels need to be handled together, the most common method is still conversion to pseudosurface models, which are inaccurate for both qualitative and quantitative evaluations. The lack of appropriate methods to visualize and analyze multichannel data compromises the benefits brought by the latest imaging techniques for fluorescence microscopy.

Since all tested software packages adopted OpenGL, an open standard for presenting 3D data, we were able to examine the implementation of each tool’s visualization pipeline, including every OpenGL function call, even for closed-source commercial tools. The visualization code programmed with certain deprecated OpenGL functions is another common drawback of all the tested tools. These functions may become unsupported in future graphics hardware drivers, making tools relying on the deprecated functions unusable. More importantly, the deprecated functions hamper the integration of potential improvements. For example, floating-point textures improve multichannel volume visualization quality, but are part of the modern OpenGL functions.

The design of FluoRender considered many-channel applications, making data loading and adjustments easier. The 96-channel test data set took less than 1 minute to load and less than 10 minutes to adjust for experienced users.

# **Supplementary Result 2. OpenCL Speed-up for Common Image Processing Filters**

We carried out a computing speed test on a Windows PC with an Intel Core i7 3.33 GHz CPU (Bloomfield) and an AMD Radeon HD7970 1 GHz GPU (Tahiti XT). The test data set is one channel of a zebrafish head confocal microscopy scan (640x640x88 voxels). It can be downloaded from FluoRender’s website (www.fluorender.org). The same set of calculations was performed on both the CPU and GPU: simple data copy, box filtering, Gaussian filtering, Sobel edge detection, median filtering, morphological erosion, and morphological dilation. The same algorithm for each filter was implemented for CPU using C++ and GPU using OpenCL. The OpenCL code has been distributed with FluoRender. The data copy simply duplicates a data set within either system memory (using the CPU) or graphics memory (using the GPU). All filters are 3D and have a window size of 3x3x3 voxels. Considering that the computing resources of the test system may be shared with other processes, we calculated the average computing time and standard error of the mean (SEM) for each filter from 10 consecutive executions.

Supplementary Table 1. OpenCL speed-up for common image processing filters.

| **Filter Type** | **Mean Time on CPU** | **Standard Err. of the Mean CPU Time** | **Mean Time on GPU** | **Standard Err. of the Mean GPU Time** | **Speedup** |
| --- | --- | --- | --- | --- | --- |
| Data copy (no filter) | 867.00 ms | 0.56% | 84.58 ms | 0.07% | 10.25x |
| Box | 4.82 s | 0.94% | 469.59 ms | 0.09% | 10.27x |
| Gaussian | 26.92 s | 3.97% | 2.41 s | 0.06% | 11.18x |
| Sobel Edge Detection | 6.97 s | 1.91% | 530.95 ms | 0.01% | 13.13x |
| Median | 20.19 s | 1.93% | 4.20 s | 0.06% | 4.81x |
| Morphological Erosion | 6.59 s | 1.42% | 471.64 ms | 0.11% | 13.97x |
| Morphological Dilation | 6.64 s | 1.39% | 469.94 ms | 0.02% | 14.14x |

All averages are calculated from 10 samples of consecutive executions of the same filter.

The computing time and GPU speed-up are listed in Supplementary Table 1. The very low standard errors signify that the results are consistent. The linear filter kernels (box and Gaussian) were evaluated on the fly for testing purposes. Therefore, the Gaussian filter, which uses exponential and power functions, took more time than the much simpler box filter. When precomputed filter kernels are used, the performance of linear filters (general convolutions) is similar to or slightly better than that of the box filter. For most common image processing filters, there is about one order of magnitude of speed-up when the GPU is utilized on the test system. The median filter has less speed-up because more conditional branches are required for sorting values. It is worth mentioning that the morphological filters (dilation and erosion) are powerful image processing tools consisting of simple computational instructions. They are also evaluated very efficiently on the GPU. Therefore, we adapted the morphological filters for real-time analysis in FluoRender.

Supplementary Video 6 demonstrates the workflow using OpenCL-based image processing filters in FluoRender. In addition to the built-in filters of FluoRender for general image processing, the integrated OpenCL kernel editor allows user customization and expansion.
